# Association of Prior Local Therapy with Outcomes Within Systemic Therapy Regimen Cohorts in Advanced Soft Tissue Sarcoma: A Retrospective Cohort Study

**DOI:** 10.3390/cancers18172806

**Published:** 2026-08-29

**Authors:** Ryotaro Ohkuma, Tomoyuki Ishiguro, Hirotsugu Ariizumi, Masahiro Shimokawa, Go Ikeda, Takahiro Yoshizawa, Yuya Hirasawa, Risako Suzuki, Toshiaki Tsurui, Emiko Mura, Rika Sasaki, Shuichi Komori, Kosuke Toyofuku, Masako Kato, Kanae Shimada, Shingo Miyamoto, Kouzou Murakami, Yoshihiro Nakagami, Kazuhiko Oshinomi, Yutaro Kubota, Hiroo Ishida, Takeshi Aoki, Masahiko Murakami, Kiyoshi Yoshimura, Satoshi Wada, Katsuhito Takahashi, Takuya Tsunoda, Atsushi Horiike

**Affiliations:** 1 Department of Oncology, Graduate School of Medicine, Showa Medical University, Tokyo 142-8555, Japan; rohkuma@med.showa-u.ac.jp (R.O.); tomo-1496@med.showa-u.ac.jp (T.I.); ariizumi@med.showa-u.ac.jp (H.A.); shimokawa.masahiro@med.showa-u.ac.jp (M.S.); gou.ikeda@showa-u.ac.jp (G.I.); takahiro.yoshizawa@showa-u.ac.jp (T.Y.); h.yuya0204@med.showa-u.ac.jp (Y.H.); risako425@med.showa-u.ac.jp (R.S.); t.tsurui@med.showa-u.ac.jp (T.T.); emikomura@med.showa-u.ac.jp (E.M.); rika.sasaki@showa-u.ac.jp (R.S.); s.komori@med.showa-u.ac.jp (S.K.); yutaro@med.showa-u.ac.jp (Y.K.); hishida@med.showa-u.ac.jp (H.I.); kyoshim1@med.showa-u.ac.jp (K.Y.); st-wada@med.showa-u.ac.jp (S.W.); ttsunoda@med.showa-u.ac.jp (T.T.); 2 Showa Medical University Comprehensive Cancer Information Center, Showa Medical University, Tokyo 142-8555, Japan; kouzou013@med.showa-u.ac.jp; 3 Division of Radiation Oncology, Department of Radiology, Graduate School of Medicine, Showa Medical University, Tokyo 142-8555, Japan; k.toyofuku@med.showa-u.ac.jp (K.T.); mkrad@med.showa-u.ac.jp (M.K.); 4 Department of Obstetrics and Gynecology, Graduate School of Medicine, Showa Medical University, Tokyo 142-8555, Japan; shimakana@med.showa-u.ac.jp (K.S.); shingo_m@med.showa-u.ac.jp (S.M.); 5 Department of Radiology, Graduate School of Medicine, Showa Medical University, Tokyo 142-8555, Japan; 6 Department of Urology, Graduate School of Medicine, Showa Medical University, Tokyo 142-8555, Japan; yo_nakagami@med.showa-u.ac.jp (Y.N.); oshikazu@med.showa-u.ac.jp (K.O.); 7 Division of Gastroenterological & General Surgery, Department of Surgery, Graduate School of Medicine, Showa Medical University, Tokyo 142-8555, Japan; takejp@med.showa-u.ac.jp (T.A.); esosurge-1@med.showa-u.ac.jp (M.M.); 8 Department of Clinical Immuno Oncology, Clinical Research Institute for Clinical Pharmacology and Therapeutics, Showa Medical University, Tokyo 157-8577, Japan; 9 Department of Clinical Diagnostic Oncology, Clinical Research Institute for Clinical Pharmacology and Therapeutics, Showa Medical University, Tokyo 157-8577, Japan; 10 Department of Sarcoma Oncology, Kameda Medical Center, Chiba 296-8602, Japan; takahashi.katsuhito@kameda.jp

**Keywords:** soft tissue neoplasms, sarcoma, neoplasm metastasis, combined modality therapy, progression-free survival, retrospective studies

## Abstract

**Background/Objectives:** Evidence regarding associations between local therapy before an indexed systemic regimen and subsequent outcomes in advanced soft tissue sarcoma (STS) remains limited. We evaluated outcomes within four regimen-defined cohorts and explored associations with prior local-therapy history. **Methods:** This retrospective cohort included 75 patients with recurrent or metastatic STS who contributed 155 treatment episodes: doxorubicin (*n* = 44), trabectedin (*n* = 31), pazopanib (*n* = 42), and eribulin (*n* = 38). Progression-free survival (PFS) and overall survival (OS) were measured from initiation of the indexed regimen. Surgery, local ablative therapy, and radiation therapy were assessed separately and in combined categories. Post hoc analyses were restricted to leiomyosarcoma. **Results:** Median PFS was 3.4, 3.1, 5.1, and 3.9 months, respectively. PFS was longer with any local therapy and surgery and/or ablation in the trabectedin cohort (*p* = 0.0342 and *p* = 0.0111) and with surgery and/or ablation in the eribulin cohort (*p* = 0.0352). Longer indexed-regimen OS was observed in surgery-containing categories in the doxorubicin and eribulin cohorts. In selected multivariable models, local therapy was associated with OS in doxorubicin- and eribulin-treated episodes and with PFS in eribulin-treated episodes. Leiomyosarcoma-restricted analyses did not consistently reproduce the full-cohort findings. **Conclusions:** Prior local-therapy history was associated with PFS or OS in selected within-cohort analyses. The associations may reflect local-treatment effects, differences among selected patients, or both; however, the study cannot distinguish these contributions. No between-regimen differences were formally tested. These findings support individualized multidisciplinary assessment and external validation.

## Simple Summary

Patients with advanced recurrent or metastatic soft tissue sarcoma may undergo selected local interventions, including surgery directed at recurrent or metastatic disease, local ablative procedures, or radiation therapy, before a subsequent systemic regimen. We analyzed 155 treatment episodes from 75 patients receiving doxorubicin, trabectedin, pazopanib, or eribulin. Prior local-therapy history was associated with longer progression-free or overall survival in selected analyses within individual regimen cohorts, whereas analyses restricted to leiomyosarcoma were less consistent. The study did not directly compare the drugs. Because the indications for local treatment and key clinical determinants of treatment selection were incompletely characterized, the observed associations may reflect clinical effects of local treatment in selected situations, differences among patients selected for such treatment, or both. These findings support individualized multidisciplinary planning and provide hypotheses for larger multicenter studies incorporating detailed disease characteristics, treatment intent, and patient-reported outcomes.

## 1. Introduction

Soft tissue sarcoma (STS) is a rare, biologically heterogeneous group of mesenchymal neoplasms that accounts for approximately 1% of adult cancers and includes more than 100 histologic subtypes [1]. This heterogeneity complicates evidence generation and treatment selection, particularly in advanced, recurrent, or metastatic disease requiring systemic therapy, in which outcomes vary with histology, disease distribution, progression rate, resectability, symptoms, and patient fitness [1,2,3,4].

Anthracycline-based treatment remains the conventional first-line therapy for most patients with advanced STS [2,3]. In the EORTC 62012 trial, intensified doxorubicin plus ifosfamide improved response rate and progression-free survival (PFS) over doxorubicin alone but conferred no clear overall survival (OS) advantage, supporting selective use when tumor shrinkage is a priority [5]. After anthracycline exposure, eribulin, pazopanib, and trabectedin are established options in appropriate histologic and clinical settings [6,7,8,9,10]. However, registration trials enroll selected populations that do not fully represent patients treated in routine practice, where histologic composition, performance status, treatment sequence, and therapeutic objectives vary more widely. Japanese and international real-world series therefore complement these data in broader clinical populations [11,12,13,14,15,16,17,18,19].

Local treatment of primary, recurrent, or metastatic sites may be considered in selected patients with advanced STS to reduce macroscopic disease, control limited metastases, relieve symptoms, or prevent local complications [2,3]. Evidence supporting surgery, local ablative procedures, and radiation therapy in this setting is predominantly retrospective and vulnerable to selection bias; favorable outcomes among locally treated patients do not establish a causal relationship with survival benefit [20,21,22,23,24,25,26,27]. These modalities also have different indications and are not equivalent interventions.

Whether a documented history of local treatment before a specific systemic therapy episode is associated with subsequent outcomes within that treatment context remains poorly understood. Because doxorubicin, trabectedin, pazopanib, and eribulin are typically used in different treatment lines and clinical settings, we evaluated outcomes within regimen-defined cohorts of patients with advanced recurrent or metastatic STS requiring systemic therapy and explored associations of prior local-therapy history—defined as local therapy performed before the indexed systemic regimen—with PFS and OS. The analysis was hypothesis-generating and was not designed to be a head-to-head comparison of systemic agents, a formal test of treatment-by-regimen interaction, or a causal evaluation of local therapy.

## 2. Materials and Methods

### 2.1. Study Design and Cohort Definition

This retrospective cohort study included patients with advanced recurrent or metastatic STS who initiated systemic therapy at Showa Medical University Hospital between April 2012 and February 2021; follow-up was updated through the March 2026 data cutoff. Eligible patients had metastatic disease at diagnosis or recurrent and/or metastatic disease after previous treatment.

Resectability was not centrally or retrospectively adjudicated at each indexed episode. Systemic therapy was initiated when the treating clinical team judged local treatment alone insufficient or inappropriate for disease control. The cohort is therefore described as having advanced recurrent or metastatic STS requiring systemic therapy, rather than as a uniformly technically unresectable population.

The source dataset comprised 75 unique patients. Principal analyses were performed at the regimen-specific treatment-episode level, with cohorts defined by treatment with doxorubicin (*n* = 44), trabectedin (*n* = 31), pazopanib (*n* = 42), or eribulin (*n* = 38). The doxorubicin cohort comprised monotherapy only; doxorubicin-containing combination regimens were excluded. The same patient could contribute to more than one regimen cohort across treatment lines; consequently, cohort sizes are not additive as unique-patient counts, and estimates across regimen cohorts are not statistically independent. The study was not designed to compare efficacy among the four agents.

Systemic therapy episodes were treatments administered at Showa Medical University Hospital. Histories of local therapy before each indexed regimen were abstracted from available clinical records, irrespective of where the procedure had been performed. Histologic diagnoses were established by two pathologists specialized in sarcoma, and the included entities are listed in Table 1. Because leiomyosarcoma was the predominant histology, post hoc sensitivity analyses were performed within that subgroup.

Sex was recorded as male or female; gender-related variables were unavailable, and sex-stratified analyses were not prespecified. Reporting was informed by the ESMO Guidance for Reporting Oncology Real-World Evidence (GROW) [28]. March 2026 denotes the cutoff for eligible clinical follow-up events, while the institutional ethics committee approved the study protocol on 20 April 2026.

### 2.2. Definition of Local Therapy Before the Indexed Systemic Regimen

For clarity, prior local therapy was defined as documented local treatment performed before initiation of the systemic regimen analyzed in that treatment episode. Three modalities were evaluated: advanced-disease-directed surgery (termed cytoreductive surgery in the analysis), local ablative therapy, and radiation therapy.

Cytoreductive surgery was operationally defined as surgery directed at recurrent or metastatic disease, performed after the diagnosis of advanced disease and before the indexed systemic regimen, with the intent of reducing or controlling macroscopic tumor burden. Initial curative-intent resection of the primary tumor before recurrent or metastatic disease and diagnostic procedures were not classified as cytoreductive surgery. Information distinguishing complete metastasectomy, incomplete debulking, palliative procedures, surgical margins, completeness of macroscopic clearance, and procedure site was not consistently available, particularly for procedures performed at outside institutions. Therefore, the variable should be interpreted as a documented history of advanced-disease-directed surgery rather than as a uniform surgical intervention.

Local ablative therapy was defined as a documented focal, nonsurgical procedure directed at a tumor lesion before initiation of the indexed systemic regimen. Radiation therapy was recorded when documented before the indexed regimen. The ablation technique, target lesion, radiation dose and fractionation, treatment intent, and whether all known disease was treated were not consistently documented. The three modalities were therefore analyzed as distinct treatment histories rather than equivalent exposures.

The three modality histories were not mutually exclusive. Two exploratory composite categories were also evaluated: (1) any local therapy (cytoreductive surgery and/or local ablative therapy and/or radiation therapy) and (2) cytoreductive surgery and/or local ablative therapy. In multivariable analyses, local therapy denoted receipt of at least one modality.

### 2.3. Outcomes and Retrospective Outcome Ascertainment

PFS was defined as the interval from initiation of the indexed systemic therapy regimen to radiologic progression according to the Response Evaluation Criteria in Solid Tumors (RECIST), version 1.1, or death from any cause, whichever occurred first; OS was defined as the interval from initiation of the indexed systemic therapy regimen to death from any cause.

Radiologic assessments were performed as part of routine clinical care at intervals set by the treating physician; no protocol-mandated imaging schedule or central radiologic review was used. Tumor response and radiologic progression were assessed by the treating physicians according to RECIST version 1.1.

Best overall response was recorded descriptively as complete response, partial response, stable disease, progressive disease, or not evaluable.

### 2.4. Statistical Analyses

Time-to-event outcomes were estimated by the Kaplan–Meier method. Within each regimen cohort, curves for episodes with and without the specified local-therapy history were compared using the log-rank test. The Gehan–Breslow–Wilcoxon test, which weights earlier events more heavily, was also reported for the full-cohort analyses.

Univariable and multivariable Cox proportional hazards models were used to explore prognostic factors. Univariable models evaluated age, sex, treatment line, performance status, and local therapy using the coding shown in Tables S3–S6. Treatment line was dichotomized as first-line versus second-line or later, and performance status as 0–1 versus ≥2; episodes with performance status 3 were included in the ≥2 category.

Because regimen-specific sample sizes and event counts were limited, each multivariable model was restricted to local therapy, including a priori as the exposure of interest, and the two variables with the lowest *p* values in the corresponding univariable analysis. Hazard ratios (HRs) and 95% confidence intervals (CIs) are reported for the second category relative to the first as coded in Tables S3–S6 (for example, performance status ≥2 versus 0–1). These models were exploratory and were not intended to estimate a causal effect or to control confounding by indication comprehensively.

The full-cohort analyses comprised 40 regimen–exposure–endpoint comparisons (four regimens × five local-therapy definitions × two endpoints). The post hoc leiomyosarcoma sensitivity analyses added 20 PFS and 20 OS log-rank comparisons. No adjustment for multiple testing was applied; *p* values should therefore be interpreted descriptively in the context of the exploratory study design. No formal interaction test was performed to assess whether associations differed among regimens.

Within-patient dependence across regimen-specific analyses was not modeled; therefore, results across regimen cohorts should not be interpreted as statistically independent comparisons.

Median follow-up was estimated separately for exposed and unexposed groups using the reverse Kaplan–Meier method and was reported as not estimable when the curve did not reach 50%. Group sizes, events, censoring, median follow-up, and numbers at risk for the full-cohort local-therapy analyses are provided in Tables S7 and S8.

All statistical tests were two-sided. Kaplan–Meier curves and survival-curve tests were generated using GraphPad Prism, version 9.4.1 (GraphPad Software, Boston, MA, USA). Cox proportional hazards analyses were performed using JMP Pro, version 17.2.0 (JMP Statistical Discovery LLC, Cary, NC, USA).

## 3. Results

### 3.1. Cohort Structure and Baseline Characteristics

The 75 unique patients contributed 155 regimen-specific treatment episodes: 44 doxorubicin, 31 trabectedin, 42 pazopanib, and 38 eribulin. Because patients could contribute across treatment lines, these counts are not additive as unique-patient counts.

Baseline clinicopathologic and treatment characteristics are summarized in Table 1. Mean age ranged from 55.38 to 59.18 years across cohorts, and most episodes involved female patients. Leiomyosarcoma was the most frequent histologic subtype; retroperitoneal and uterine primary sites predominated; and performance status 1 was most frequent at regimen initiation. Doxorubicin was used mainly as first-line therapy (32 of 44 episodes); trabectedin and eribulin were concentrated in later lines, whereas pazopanib had a mixed line-of-therapy distribution. Histories of cytoreductive surgery, local ablative therapy, and radiation therapy were common and overlapping. Stable disease was the most frequent best overall response in each cohort (Table 1).

### 3.2. Regimen-Specific PFS

Median PFS was 3.4 months with doxorubicin, 3.1 months with trabectedin, 5.1 months with pazopanib, and 3.9 months with eribulin (Figure S1).

The leiomyosarcoma subgroup comprised 25 doxorubicin-, 21 trabectedin-, 21 pazopanib-, and 23 eribulin-treated episodes. Median PFS within this subgroup was 4.6, 3.1, 5.0, and 5.1 months, respectively (Figure S1).

### 3.3. PFS According to Prior Local-Therapy History

**Figure 1:**
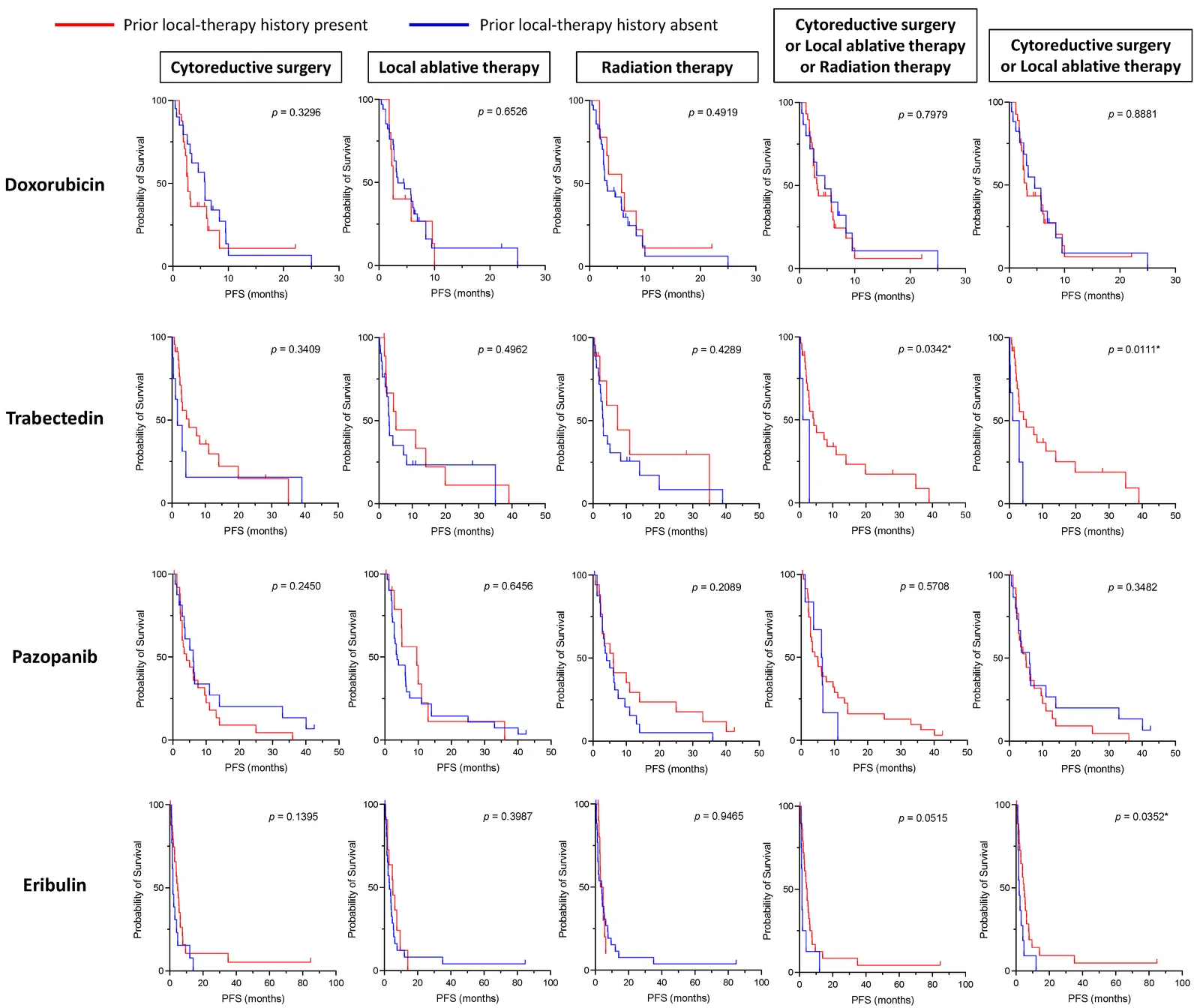
Progression-free survival according to prior local-therapy history by systemic therapy regimen. Kaplan–Meier estimates of progression-free survival (PFS) in regimen-specific treatment episodes treated with doxorubicin, trabectedin, pazopanib, or eribulin. PFS was measured from the initiation of the indexed systemic therapy regimen to radiologic progression or death, whichever occurred first. Rows correspond to systemic therapy regimens, and columns correspond to the following prior local-therapy categories: cytoreductive surgery; local ablative therapy; radiation therapy; cytoreductive surgery and/or local ablative therapy and/or radiation therapy; cytoreductive surgery and/or local ablative therapy. The five local-therapy definitions were evaluated separately and were not mutually exclusive across panels. Red lines indicate the presence of the specified local-therapy history, and blue lines indicate its absence. Two-sided log-rank tests were used for between-group comparisons; corresponding Gehan–Breslow–Wilcoxon results are presented in Table S1. Group sizes, PFS events, censoring, median follow-up, and numbers at risk are reported in Table S7. No adjustment for multiple comparisons was performed; *p* values should be interpreted in the exploratory context of the study. Tick marks indicate censored observations. * *p* < 0.05.

Kaplan–Meier analyses of PFS according to prior local-therapy history are shown in Figure 1. Corresponding log-rank and Gehan–Breslow–Wilcoxon *p* values are provided in Table S1, and group sizes, PFS events, censoring, median follow-up, and numbers at risk are provided in Table S7.

No local-therapy comparison had a log-rank *p* value below 0.05 in the doxorubicin or pazopanib cohorts.

In the trabectedin cohort, any local therapy was associated with longer PFS (log-rank *p* = 0.0342), as was cytoreductive surgery and/or local ablative therapy (*p* = 0.0111). Cytoreductive surgery alone was not associated with PFS by the log-rank test (*p* = 0.3409), although the Gehan–Breslow–Wilcoxon *p* value was 0.0491, suggesting that the observed curve separation was weighted toward earlier events (Figure 1; Tables S1 and S7).

In the eribulin cohort, cytoreductive surgery and/or local ablative therapy were associated with longer PFS (log-rank *p* = 0.0352). For any local therapy, the log-rank *p* value was 0.0515, whereas the Gehan–Breslow–Wilcoxon *p* value was 0.0165. Radiation therapy alone was not associated with PFS (log-rank *p* = 0.9465) (Figure 1; Tables S1 and S7).

These within-cohort findings do not establish whether associations differed among regimens because no formal interaction test was performed.

In the post hoc leiomyosarcoma sensitivity analyses, none of the 20 local-therapy comparisons had a log-rank *p* value below 0.05 (Figure S2). Thus, the full-cohort PFS associations were not consistently reproduced within the predominant histologic subgroup.

### 3.4. OS According to Prior Local-Therapy History

**Figure 2:**
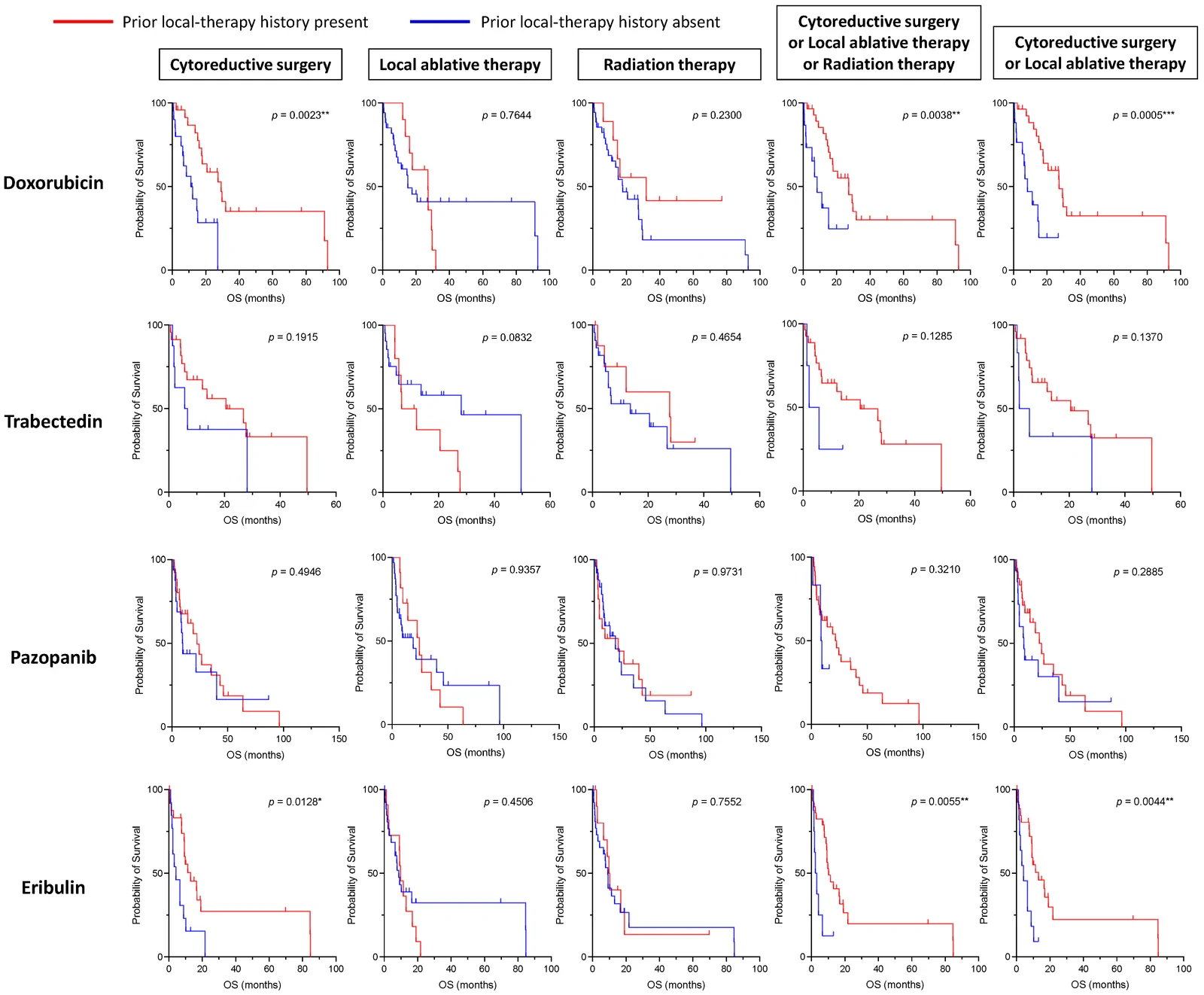
Overall survival according to prior local-therapy history by systemic therapy regimen. Kaplan–Meier estimates of overall survival (OS) in regimen-specific treatment episodes treated with doxorubicin, trabectedin, pazopanib, or eribulin. OS was measured from initiation of the indexed systemic therapy regimen to death from any cause. Rows correspond to systemic therapy regimens, and columns correspond to the following prior local-therapy categories: cytoreductive surgery, local ablative therapy, radiation therapy, cytoreductive surgery and/or local ablative therapy and/or radiation therapy, and cytoreductive surgery and/or local ablative therapy. The five local-therapy definitions were evaluated separately and were not mutually exclusive across panels. Red lines indicate the presence of the specified local-therapy history, and blue lines indicate its absence. Two-sided log-rank tests were used for between-group comparisons; corresponding Gehan–Breslow–Wilcoxon results are presented in Table S2. Group sizes, deaths, censoring, median follow-up, and numbers at risk are reported in Table S8. No adjustment for multiple comparisons was performed; *p* values should be interpreted in the exploratory context of the study. Tick marks indicate censored observations. * *p* < 0.05; ** *p* < 0.01; *** *p* < 0.001.

OS was measured from initiation of each indexed systemic regimen. Kaplan–Meier analyses according to prior local-therapy history are shown in Figure 2; corresponding log-rank and Gehan–Breslow–Wilcoxon *p* values are provided in Table S2; and group sizes, deaths, censoring, median follow-up, and numbers at risk are provided in Table S8.

In the doxorubicin cohort, cytoreductive surgery was associated with longer OS (log-rank *p* = 0.0023), as was any local therapy (*p* = 0.0038) and cytoreductive surgery and/or local ablative therapy (*p* = 0.0005). Local ablative therapy alone and radiation therapy alone were not associated with OS (Figure 2; Tables S2 and S8).

In the trabectedin cohort, none of the five local-therapy comparisons had a log-rank *p* value below 0.05; *p* values ranged from 0.0832 to 0.4654 (Figure 2; Tables S2 and S8).

In the pazopanib cohort, none of the five local-therapy comparisons had a log-rank *p* value below 0.05; *p* values ranged from 0.2885 to 0.9731. The corresponding Gehan–Breslow–Wilcoxon *p* values were also all greater than 0.05 (Figure 2; Tables S2 and S8).

In the eribulin cohort, cytoreductive surgery was associated with longer OS (*p* = 0.0128), as were any local therapies (*p* = 0.0055) and cytoreductive surgery and/or local ablative therapy (*p* = 0.0044). Local ablative therapy alone and radiation therapy alone were not associated with OS (Figure 2; Tables S2 and S8).

In post hoc analyses restricted to leiomyosarcoma, only the doxorubicin comparison of cytoreductive surgery and/or local ablative therapy had a *p* value below 0.05 (*p* = 0.0302); the other 19 comparisons had *p* values greater than 0.05 (Figure S3). These subgroup findings should be interpreted cautiously because of small exposure groups and multiple testing.

### 3.5. Exploratory Prognostic-Factor Analyses

Because local therapy was the exposure of interest, the summary below focuses on its estimates. All covariate estimates are provided in Tables S3–S6. The multivariable models adjusted only for selected available covariates and were not intended to eliminate confounding by indication.

In doxorubicin-treated episodes, local therapy was not associated with PFS in the selected multivariable model (HR, 1.01; 95% CI, 0.47–2.18; *p* = 0.97), but it was associated with longer indexed-regimen OS (HR, 0.22; 95% CI, 0.079–0.60; *p* = 0.0029) (Table S3).

In trabectedin-treated episodes, local therapy was not associated with PFS after adjustment for selected available covariates (HR, 0.52; 95% CI, 0.13–2.04; *p* = 0.35) or with indexed-regimen OS (*p* = 0.32) (Table S4).

In pazopanib-treated episodes, local therapy was not associated with PFS (HR, 0.70; 95% CI, 0.28–1.75; *p* = 0.44) or indexed-regimen OS (HR, 0.44; 95% CI, 0.14–1.42; *p* = 0.17) in the selected multivariable models (Table S5).

In eribulin-treated episodes, local therapy was associated with longer PFS (HR, 0.30; 95% CI, 0.10–0.91; *p* = 0.034) and longer index-regimen OS (HR, 0.29; 95% CI, 0.097–0.86; *p* = 0.025) after adjustment for selected available covariates (Table S6).

Given the data-driven covariate selection, small regimen cohorts, limited event counts, multiple analyses, and the absence of major clinical confounders from the models, all adjusted estimates remain exploratory.

## 4. Discussion

This study provides a within-regimen view of how local-treatment history relates to subsequent systemic-treatment outcomes in advanced STS. Two findings stand out. First, PFS was longer in selected trabectedin- and eribulin-treated episodes with a history of local therapy, most clearly in categories including surgery and/or local ablation. Second, longer OS was observed in surgery-containing categories in the doxorubicin and eribulin cohorts. These patterns neither rank the systemic agents nor demonstrate a treatment-by-regimen interaction, but they suggest that prior local-therapy history carries clinically relevant information about the disease course and the multidisciplinary treatment trajectory of patients entering an indexed regimen.

The regimen-specific PFS estimates were broadly consistent with the range reported in pivotal trials and real-world studies. Median PFS with doxorubicin was shorter than the 4.6 months reported with first-line doxorubicin in EORTC 62012, plausibly reflecting the broader histologic composition and inclusion of later-line episodes in this cohort [5]. Trabectedin PFS was shorter than in the pivotal phase III trabectedin trial but within the range described in subsequent real-world series [10,17,18]. Pazopanib PFS was similar to that in PALETTE and later real-world studies [9,15,16,19]. Eribulin PFS, including the leiomyosarcoma estimate, appeared longer than in the randomized leiomyosarcoma subgroup; nevertheless, randomized evidence shows the clearest eribulin survival advantage in liposarcoma rather than leiomyosarcoma [6,7,8]. These comparisons support the clinical plausibility of the observed outcomes while underscoring that this leiomyosarcoma-rich cohort cannot establish histology-specific superiority.

Associations were observed in selected analyses within the trabectedin and eribulin cohorts. One possible explanation relates to the different clinical settings in which the regimens were used. Doxorubicin was used predominantly first-line, when disease burden, symptoms, and the need for rapid tumor shrinkage may dominate early outcome, whereas pazopanib was used across mixed lines, often as an oral disease-control strategy. By contrast, trabectedin and eribulin were concentrated in later-line, leiomyosarcoma-rich trajectories, in which patients had already undergone repeated multidisciplinary assessment. In that setting, previous surgery or ablation may have reduced active tumor burden, making subsequent disease control more readily observable. Alternatively, eligibility for local therapy may identify patients with favorable anatomy, slower progression, preserved performance status, and continued access to specialist care. These hypotheses may contextualize the observed within-cohort patterns, but no formal interaction test was performed; differences in *p* values across cohorts therefore cannot establish that the association differed among systemic regimens.

The pattern across local-treatment categories is also informative: associations were generally more evident in combined categories containing surgery than with radiation therapy alone. Surgery and local ablation are typically selected when macroscopic disease reduction or control of a limited site is technically feasible, whereas radiation therapy in advanced STS may serve wider purposes, including palliation, prevention of local complications, and ablative treatment of limited disease. Each modality should therefore be interpreted within its clinical context, not as an equivalent local treatment. Aligning the OS origin with that for PFS excludes survival time accrued before the indexed regimen and strengthens temporal coherence.

The observed associations may reflect a combination of clinical effects of local treatment in selected situations and systematic differences among patients selected for such treatment. Local interventions may contribute to local disease control, symptom relief, or prevention of local complications, whereas patients eligible for these interventions may also have lower disease burden, more favorable anatomy, slower disease progression, or better-preserved fitness. Because these determinants were incompletely characterized in the retrospective records, the present study cannot distinguish between the relative contributions of treatment and selection.

The post hoc leiomyosarcoma analyses examined whether the overall findings were driven by the predominant histology; the fact that none of the PFS comparisons and only one OS comparison reached *p* < 0.05 tempers any histology-specific interpretation. The substantially smaller exposure groups also reduce precision, so these results should not be viewed as disproving the full-cohort associations but rather as reinforcing the need to evaluate treatment sequence within larger, histologically defined datasets.

The findings fit the individualized approach of contemporary multidisciplinary sarcoma practice. Guidelines support selective use of surgery, ablative procedures, and radiation therapy while recognizing that randomized evidence for a survival advantage in metastatic STS is lacking [2,3]. Systematic reviews of pulmonary metastasectomy and reports of ablative treatment describe durable local control and prolonged survival in carefully selected patients, although heterogeneous indications and confounding by selection remain important [20,21,22,23,24,25,26,27,29,30]. Samà et al. similarly emphasized integrating surgical and nonsurgical strategies according to the clinical scenario through multidisciplinary decision-making at experienced referral centers [31]. Decisions regarding the indication, integration, and sequencing of surgery, local ablative procedures, radiation therapy, and systemic treatment should be individualized through multidisciplinary discussion involving medical oncology, radiation oncology, surgery, and other relevant specialists. The present study contributes a treatment-sequence perspective but does not establish a preferred local modality or systemic regimen.

Several features strengthen this study: long-term follow-up updated through March 2026, routine-practice treatment sequences that are difficult to represent in prospective trials, and evaluation of three local-treatment modalities across four widely used regimens. The treatment-episode design reflects the longitudinal nature of advanced STS care, and distinguishing 75 unique patients from 155 treatment episodes avoids presenting regimen cohorts as independent patient populations.

The study nevertheless has important limitations. It was retrospective and hospital-based, with modest regimen-specific sample sizes, heterogeneous histologies and treatment lines, a predominance of leiomyosarcoma and uterine or retroperitoneal primaries, and some entities falling outside conventional malignant adult-type STS classifications. Patients could contribute to more than one regimen cohort, and within-patient correlation was not modeled; estimates across cohorts are therefore not statistically independent, and their precision may be overstated. Detailed data on metastatic burden and distribution, synchronous versus metachronous presentation, disease-free interval, progression kinetics, resectability, treatment intent, completeness of resection, ablation technique, radiation dose, and local-treatment timing were not consistently available; because these factors influence both prognosis and selection for local therapy, substantial potential remains for confounding by indication, exposure misclassification, and lead- or length-time bias. Multiple comparisons were also performed without adjustment; the multivariable models were limited by event counts and data-driven covariate selection; no formal interaction test was undertaken; and imaging was assessed in routine practice without central review. Patient-reported outcomes, symptom-control measures, functional outcomes, and quality-of-life data were not collected; future studies should incorporate these endpoints, particularly when evaluating local interventions performed with palliative intent. The results should therefore be interpreted as hypothesis-generating associations rather than causal estimates.

Despite these constraints, this study identifies a clinically relevant signal across sequential local and systemic treatment settings in a rare and heterogeneous disease. Multicenter studies with standardized characterization of metastatic phenotype and local-treatment intent, patient-level methods that account for repeated episodes, patient-reported outcomes, and prespecified analyses of histology and treatment context are warranted to clarify which patients might derive the greatest value from integrated multidisciplinary strategies.

## 5. Conclusions

In this exploratory treatment-episode cohort of patients with advanced recurrent or metastatic STS requiring systemic therapy, prior local-therapy history was associated with longer PFS or OS in selected within-regimen analyses. The observed associations may reflect clinical effects of local treatment in selected situations, differences among patients selected for such treatment, or both; the present data cannot distinguish these contributions, and no between-regimen differences were formally tested. Nevertheless, the findings provide a clinically relevant treatment-sequence perspective and support individualized multidisciplinary assessment of how local and systemic modalities are integrated. Validation in larger multicenter studies with detailed disease characteristics, treatment intent, patient-reported outcomes, and patient-level longitudinal methods is warranted.

## Figures and Tables

**Table 1:** Clinicopathological and treatment characteristics of regimen-specific treatment episodes in advanced, recurrent, or metastatic soft tissue sarcoma requiring systemic therapy.

Characteristic	Doxorubicin *n* = 44	Trabectedin *n* = 31	Pazopanib *n* = 42	Eribulin *n* = 38
Age, mean (standard deviation), years	59.18 ± 13.28	55.38 ± 13.22	56.26 ± 14.50	58.32 ± 13.18
Sex (*n*)				
Male	12	6	12	9
Female	32	25	30	29
Histological type (*n*)				
Leiomyosarcoma	25	21	21	23
Liposarcoma	6	3	1	4
Unclassifiable	4	4	2	3
Angiosarcoma	0	0	4	1
Spindle cell sarcoma	4	0	2	0
Carcinosarcoma	1	2	3	2
Desmoid-type fibromatosis	1	0	1	0
Malignant phyllodes tumor	1	1	3	3
Myxofibrosarcoma	1	0	0	0
Undifferentiated pleomorphic sarcoma	1	0	1	1
Epithelioid hemangioendothelioma	0	0	1	0
Endometrial stromal sarcoma	0	0	1	0
Intimal sarcoma	0	0	1	0
Histiocytic sarcoma	0	0	1	1
Primary site (*n*)				
Retroperitoneum	22	13	11	12
Uterus	9	10	12	13
Genitourinary tract	4	4	3	3
Lung	2	0	2	0
Liver	2	1	1	1
Breast	1	1	3	3
Thigh	1	0	0	0
Gastrointestinal	1	2	2	3
Head and neck	0	0	4	1
Cardiovascular	0	0	2	1
Spleen	0	0	1	1
Adrenal gland	0	0	1	0
Treatment line (*n*)				
1st	32	1	15	3
2nd	10	10	18	14
3rd	2	14	4	14
4th	0	6	5	5
5th	0	0	0	2
Performance status at initiation of systemic chemotherapy (*n*)				
0	5	3	2	0
1	31	22	33	31
2	7	6	4	6
3	1	0	3	1
Best overall response (*n*)				
CR	0	0	1	1
PR	4	4	6	4
SD	24	14	21	17
PD	16	13	14	15
NE	0	0	0	1
History of cytoreductive surgery (*n*)				
Yes	24	23	26	25
No	20	8	16	13
History of local ablative therapy (*n*)				
Yes	10	10	11	11
No	34	21	31	27
History of radiation therapy (*n*)				
Yes	9	9	17	11
No	35	22	25	27

Values are the numbers of treatment episodes unless otherwise indicated. Local-therapy categories were evaluated separately and were not mutually exclusive. CR, complete response; NE, not evaluable; PD, progressive disease; PR, partial response; SD, stable disease.

## Data Availability

Deidentified data underlying the analyses are available from the corresponding author upon reasonable request, subject to institutional ethics and privacy restrictions.

## Supplementary Materials

The following supporting information can be downloaded at https://www.mdpi.com/article/10.3390/cancers18172806/s1, Figure S1: Kaplan–Meier Curves for Progression-Free Survival According to Systemic Therapy Regimen in the Overall Cohort and the Leiomyosarcoma Subgroup; Figure S2: Progression-Free Survival According to Prior Local-Therapy History by Systemic Therapy Regimen in the Leiomyosarcoma Subgroup; Figure S3: Overall Survival According to Prior Local-Therapy History by Systemic Therapy Regimen in the Leiomyosarcoma Subgroup; Table S1: Comparison of Log-Rank and Gehan–Breslow–Wilcoxon *p* Values for Progression-Free Survival According to Prior Local-Therapy Category by Systemic Therapy Regimen; Table S2: Comparison of Log-Rank and Gehan–Breslow–Wilcoxon *p* Values for Overall Survival According to Prior Local-Therapy Category by Systemic Therapy Regimen; Table S3: Univariable and Multivariable Analyses of Prognostic Factors for Progression-Free and Overall Survival in Doxorubicin-Treated Episodes; Table S4: Univariable and Multivariable Analyses of Prognostic Factors for Progression-Free and Overall Survival in Trabectedin-Treated Episodes; Table S5: Univariable and Multivariable Analyses of Prognostic Factors for Progression-Free and Overall Survival in Pazopanib-Treated Episodes; Table S6: Univariable and Multivariable Analyses of Prognostic Factors for Progression-Free and Overall Survival in Eribulin-Treated Episodes; Table S7: Numbers at Risk, Events, and Follow-Up for Progression-Free Survival Analyses According to Prior Local-Therapy History by Systemic Therapy Regimen; Table S8: Numbers at Risk, Deaths, and Follow-Up for Overall Survival Analyses According to Prior Local-Therapy History by Systemic Therapy Regimen.

## Abbreviations

The following abbreviations are used in this manuscript:

CI	Confidence intervals
HR	Hazard ratios
OS	Overall survival
PFS	Progression-free survival
RECIST	Response Evaluation Criteria in Solid Tumors
SD	Stable disease
STS	Soft tissue sarcoma

## References

[1] Gamboa A.C., Gronchi A., Cardona K. (2020). Soft-Tissue Sarcoma in Adults: An Update on the Current State of Histiotype-Specific Management in an Era of Personalized Medicine. CA Cancer J. Clin..

[2] Gronchi A., Miah A.B., Dei Tos A.P., Abecassis N., Bajpai J., Bauer S., Biagini R., Bielack S., Blay J.Y., Bolle S. (2021). EURACAN and GENTURIS. Soft Tissue and Visceral Sarcomas: ESMO-EURACAN-GENTURIS Clinical Practice Guidelines for Diagnosis, Treatment and Follow-Up. Ann. Oncol..

[3] von Mehren M., Kane J.M., Armstrong S.A., Balach T., Bishop A.J., Buehler D., Carr-Ascher J., Choy E., Cipriano C., Connolly M. (2025). NCCN Guidelines^®^ Insights: Soft Tissue Sarcoma, Version 1.2025. J. Natl. Compr. Cancer Netw..

[4] In G.K., Hu J.S., Tseng W.W. (2017). Treatment of Advanced, Metastatic Soft Tissue Sarcoma: Latest Evidence and Clinical Considerations. Ther. Adv. Med. Oncol..

[5] Judson I., Verweij J., Gelderblom H., Hartmann J.T., Schöffski P., Blay J.Y., Kerst J.M., Sufliarsky J., Whelan J., Hohenberger P. (2014). Doxorubicin Alone Versus Intensified Doxorubicin plus Ifosfamide for First-Line Treatment of Advanced or Metastatic Soft-Tissue Sarcoma: A Randomised Controlled Phase 3 Trial. Lancet Oncol..

[6] Schöffski P., Chawla S., Maki R.G., Italiano A., Gelderblom H., Choy E., Grignani G., Camargo V., Bauer S., Rha S.Y. (2016). Eribulin Versus Dacarbazine in Previously Treated Patients with Advanced Liposarcoma or Leiomyosarcoma: A Randomised, Open-Label, Multicentre, Phase 3 Trial. Lancet.

[7] Blay J.Y., Schöffski P., Bauer S., Krarup-Hansen A., Benson C., D’Adamo D.R., Jia Y., Maki R.G. (2019). Eribulin Versus Dacarbazine in Patients with Leiomyosarcoma: Subgroup Analysis from a Phase 3, Open-Label, Randomised Study. Br. J. Cancer.

[8] Demetri G.D., Schöffski P., Grignani G., Blay J.Y., Maki R.G., Van Tine B.A., Alcindor T., Jones R.L., D’Adamo D.R., Guo M. (2017). Activity of Eribulin in Patients with Advanced Liposarcoma Demonstrated in a Subgroup Analysis From a Randomized Phase III Study of Eribulin Versus Dacarbazine. J. Clin. Oncol..

[9] van der Graaf W.T.A., Blay J.Y., Chawla S.P., Kim D.W., Bui-Nguyen B., Casali P.G., Schöffski P., Aglietta M., Staddon A.P., Beppu Y. (2012). Pazopanib for Metastatic Soft-Tissue Sarcoma (PALETTE): A Randomised, Double-Blind, Placebo-Controlled Phase 3 Trial. Lancet.

[10] Demetri G.D., von Mehren M., Jones R.L., Hensley M.L., Schuetze S.M., Staddon A., Milhem M., Elias A., Ganjoo K., Tawbi H. (2016). Efficacy and Safety of Trabectedin or Dacarbazine for Metastatic Liposarcoma or Leiomyosarcoma After Failure of Conventional Chemotherapy: Results of a Phase III Randomized Multicenter Clinical Trial. J. Clin. Oncol..

[11] Nakamura T., Asanuma K., Hagi T., Sudo A. (2020). Clinical Outcome of Systemic Treatment for Advanced Soft Tissue Sarcoma: Real-Life Perspective in Japan. Drug Des. Dev. Ther..

[12] Nakamura T., Tsukushi S., Asanuma K., Katagiri H., Ikuta K., Nagano A., Kozawa E., Yamada S., Shido Y., Yamada K. (2019). The Clinical Outcome of Eribulin Treatment in Japanese Patients with advanced soft tissue sarcoma: A Tokai Musculoskeletal Oncology Consortium Study. Clin. Exp. Metastasis.

[13] Kobayashi E., Naito Y., Asano N., Maejima A., Endo M., Takahashi S., Megumi Y., Kawai A. (2019). Interim results of a real-world observational study of eribulin in soft tissue sarcoma including rare subtypes. Jpn. J. Clin. Oncol..

[14] Kawai A., Narahara H., Takahashi S., Nakamura T., Kobayashi H., Megumi Y., Matsuoka T., Kobayashi E. (2022). Safety and effectiveness of eribulin in Japanese patients with soft tissue sarcoma including rare subtypes: A post-marketing observational study. BMC Cancer.

[15] Oh C.R., Hong J.Y., Kim J.H., Lee J.S., Kim H.S., Kim T.W., Ahn J.H., Kim J.E. (2020). Real-World Outcomes of Pazopanib Treatment in Korean Patients with Advanced Soft Tissue Sarcoma: A Multicenter Retrospective Cohort Study. Target. Oncol..

[16] Bilici A., Koca S., Karaagac M., Aydin S.G., Eraslan E., Kaplan M.A., Ocak B., Goksu S.S., Paydas S., Akgul F. (2023). Real-World Outcomes of Pazopanib in Metastatic Soft Tissue Sarcoma: A Retrospective Turkish Oncology Group (TOG) Study. J. Cancer Res. Clin. Oncol..

[17] Vincenzi B., Napolitano A., Comandone A., Sanfilippo R., Celant S., Olimpieri P.P., Di Segni S., Russo P., Casali P.G. (2023). Trabectedin use in soft-tissue sarcoma patients in a real-world setting: Data from an Italian National Drug-Access Registry. Int. J. Cancer.

[18] Chaigneau L., Jary M., Nerich V., Hervieu A., Aubry S., Charon Barra C., Meynard G., Neumann F., Kalbacher E., Isambert N. (2022). Real-World Experience of Efficacy and Safety of Trabectedin in Patients with Soft Tissue Sarcoma: A Bicentric Retrospective Analysis. Oncology.

[19] Vincenzi B., Olimpieri P.P., Celant S., Mazzocca A., Cortellini A., Comandone A., Tomassini L., Di Segni S., Russo P., Casali P.G. (2024). Pazopanib in the real-world setting in soft tissue sarcomas: Data from the Italian national registry. ESMO Open.

[20] Treasure T., Fiorentino F., Scarci M., Møller H., Utley M. (2012). Pulmonary Metastasectomy for Sarcoma: A Systematic Review of Reported Outcomes in the Context of Thames Cancer Registry Data. BMJ Open.

[21] Stamenovic D., Hohenberger P., Roessner E. (2021). Pulmonary metastasectomy in soft tissue sarcomas: A systematic review. J. Thorac. Dis..

[22] Gronchi A., Guadagnolo B.A., Erinjeri J.P. (2016). Local Ablative Therapies to Metastatic Soft Tissue Sarcoma. Am. Soc. Clin. Oncol. Educ. Book.

[23] Shah N.K., Yegya-Raman N., Jones J.A., Shabason J.E. (2021). Radiation Therapy in Metastatic Soft Tissue Sarcoma: From Palliation to Ablation. Cancers.

[24] Feng X.Y., Li J., Li A.M., Jing S.H., Zhu X.X., Wang Z. (2022). Stereotactic body radiotherapy for recurrent and oligometastatic soft tissue sarcoma. World J. Surg. Oncol..

[25] Burkhard-Meier A., Grube M., Jurinovic V., Agaimy A., Albertsmeier M., Berclaz L.M., Di Gioia D., Dürr H.R., von Eisenhart-Rothe R., Eze C. (2024). Unraveling the role of local ablative therapies for patients with metastatic soft tissue sarcoma—A retrospective multicenter study of the Bavarian university hospitals. Eur. J. Surg. Oncol..

[26] Llacer-Moscardo C., Moureau-Zabotto L., Ollivier L., Helfré S., Ducassou A., Bonvalot S., Sunyach M.P., Sargos P., Gillon P., Firmin N. (2024). Management of oligometastatic/metastatic sarcomas and place of local treatments with focus on modern radiotherapy approaches. Cancer Radiother..

[27] Bonvalot S., Tetreau R., Llacer-Moscardo C., Roland C. (2024). The Landmark Series: Multimodal Management of Oligometastatic Sarcoma. Ann. Surg. Oncol..

[28] Castelo-Branco L., Pellat A., Martins-Branco D., Valachis A., Derksen J.W.G., Suijkerbuijk K.P.M., Dafni U., Dellaporta T., Vogel A., Prelaj A. (2023). ESMO Guidance for Reporting Oncology real-World evidence (GROW). Ann. Oncol..

[29] Hedman M., Rossi E., Dalqvist E., Karlsson K., Linder-Stragliotto C. (2025). Improved local control using higher dose SBRT in metastatic sarcoma patients. Radiat. Oncol..

[30] Kretzschmar L., Fritsak M., Heesen P., Heusel A., Bonvalot S., Guckenberger M., Miah A., Röder F., Smolle M.A., Christ S.M. (2025). Stereotactic Body Radiotherapy vs. Metastasectomy for Soft Tissue and Bone Sarcoma Lung Metastases—A Systematic Review analyzing Safety and Efficacy. Clin. Transl. Radiat. Oncol..

[31] Samà L., Rodda G.A., Ruspi L., Sicoli F., D’Amato V., Renne S.L., Laffi A., Baldaccini D., Clerici E., Navarria P. (2024). Mesenchymal Tumor Management: Integrating Surgical and Non-Surgical Strategies in Different Clinical Scenarios. Cancers.

