# Supplementary material for: Association of Prior Local Therapy with Outcomes Within Systemic Therapy Regimen Cohorts in Advanced Soft Tissue Sarcoma: A Retrospective Cohort Study
Source: Cancers (Basel). 2026 Aug 29;18(17):2806. doi: 10.3390/cancers18172806 (PMC13565411; doi:10.3390/cancers18172806)
Supplement: Supplementary file 1 [file cancers-18-02806-s001.zip › cancers-4498027-supplementary.pdf]

Supplementary Materials

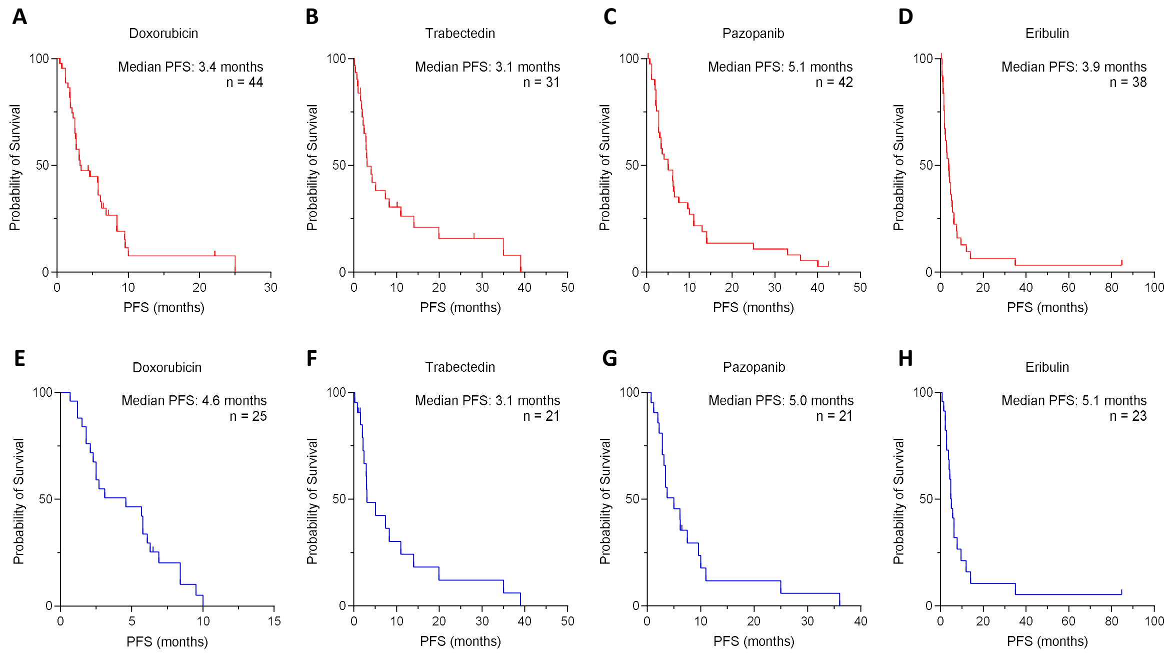

Figure S1. Kaplan-Meier Curves for Progression-Free Survival According to Systemic Therapy Regimen in the Overall Cohort and the Leiomyosarcoma Subgroup

Panels A-D show PFS in the overall regimen-specific cohorts receiving doxorubicin (A), trabectedin (B), pazopanib (C), and eribulin (D). Panels E-H show the corresponding leiomyosarcoma subgroup analyses for doxorubicin (E), trabectedin (F), pazopanib (G), and eribulin (H). In the overall cohort, median PFS was 3.4 months with doxorubicin (n = 44), 3.1 months with trabectedin (n = 31), 5.1 months with pazopanib (n = 42), and 3.9 months with eribulin (n = 38). In the leiomyosarcoma subgroup, median PFS was 4.6 months with doxorubicin (n = 25), 3.1 months with trabectedin (n = 21), 5.0 months with pazopanib (n = 21), and 5.1 months with eribulin (n = 23). Tick marks indicate censored observations. PFS, progression-free survival.

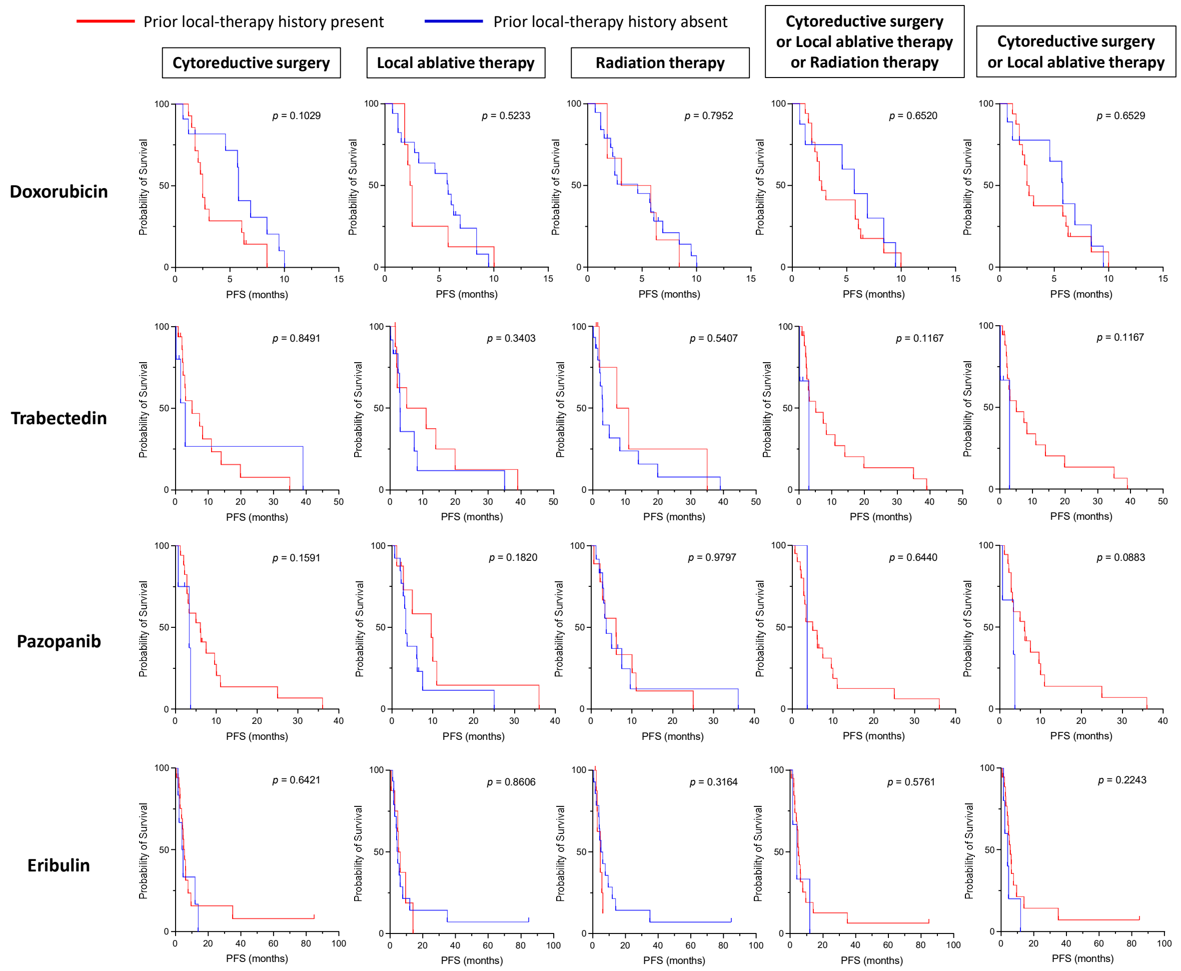

Figure S2. Progression-Free Survival According to Prior Local-Therapy History by Systemic Therapy Regimen in the Leiomyosarcoma Subgroup

Progression-Free Survival According to Prior Local-Therapy History by Systemic Therapy Regimen in the Leiomyosarcoma Subgroup. Kaplan–Meier estimates of PFS among leiomyosarcoma treatment episodes treated with doxorubicin (n = 25), trabectedin (n = 21), pazopanib (n = 21), or eribulin (n = 23). PFS was measured from initiation of the indexed systemic therapy regimen to radiologic progression according to Response Evaluation Criteria in Solid Tumors, version 1.1, or death from any cause, whichever occurred first. Rows correspond to systemic therapy regimens, and columns correspond to the five prior local-therapy categories used in the full-cohort analyses. The in-figure key identifies red lines as prior local-therapy history present and blue lines as absent. Two-sided log-rank tests were used. These post hoc sensitivity analyses were exploratory. No adjustment for multiple comparisons was performed, and none of the 20 comparisons had a p value below 0.05. Tick marks indicate censored observations.

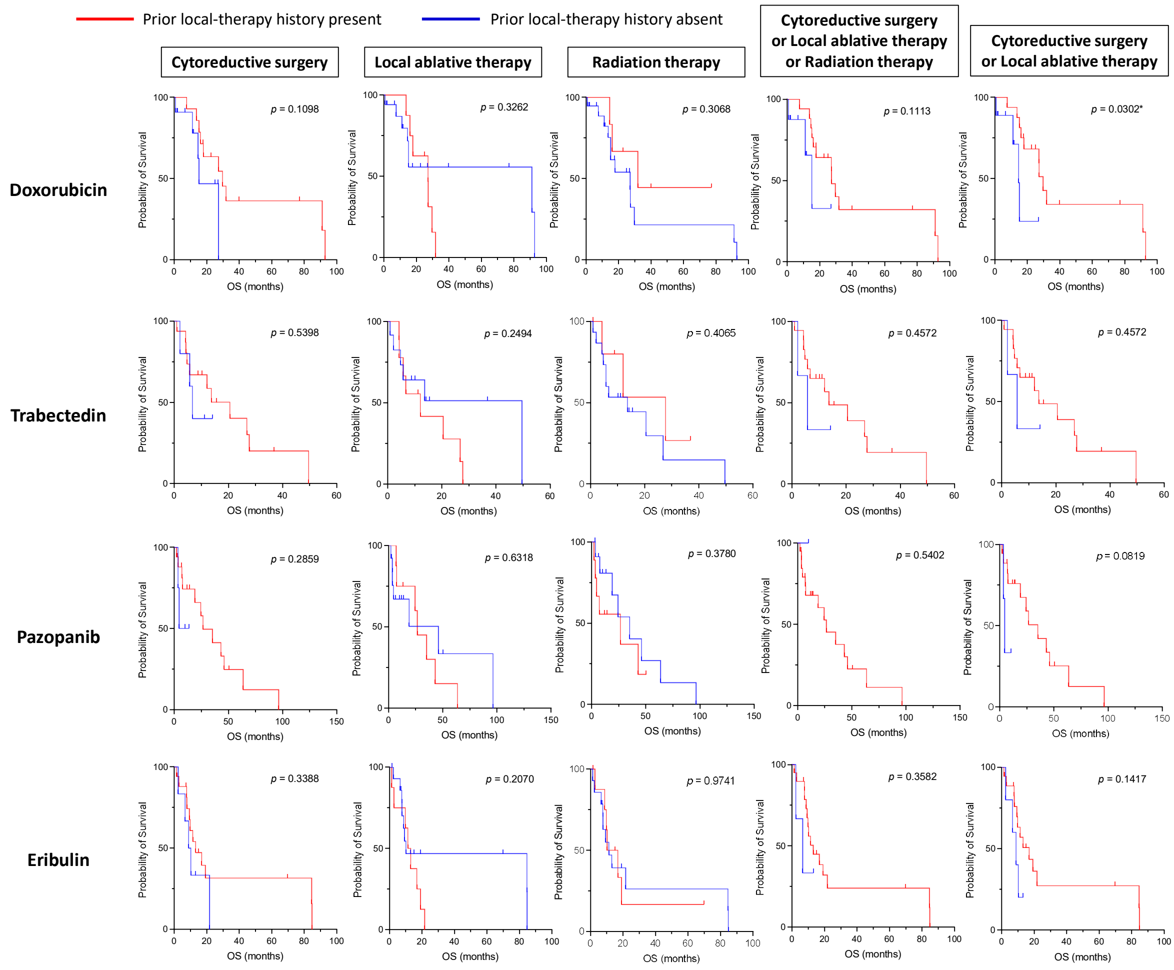

Figure S3. Overall Survival According to Prior Local-Therapy History by Systemic Therapy Regimen in the Leiomyosarcoma Subgroup

Overall Survival According to Prior Local-Therapy History by Systemic Therapy Regimen in the Leiomyosarcoma Subgroup. Kaplan–Meier estimates of OS among leiomyosarcoma treatment episodes treated with doxorubicin (n = 25), trabectedin (n = 21), pazopanib (n = 21), or eribulin (n = 23). OS was measured from initiation of the indexed systemic therapy regimen to death from any cause. Rows correspond to systemic therapy regimens, and columns correspond to the five prior local-therapy categories used in the full-cohort analyses. The in-figure key identifies red lines as prior local-therapy history present and blue lines as absent. Two-sided log-rank tests were used. These post hoc sensitivity analyses were exploratory, and no adjustment for multiple comparisons was performed. Only the doxorubicin comparison for cytoreductive surgery and/or local ablative therapy had a p value below 0.05 (p = 0.0302); the other 19 comparisons had p values greater than 0.05. Tick marks indicate censored observations. *p < 0.05.

Table S1. Comparison of Log-Rank and Gehan–Breslow–Wilcoxon p Values for Progression-Free Survival According to Prior Local-Therapy Category by Systemic Therapy Regimen

Systemic therapy regimen	Treatment category	P-value for PFS	
		Log-rank test	Gehan–Breslow–Wilcoxon test
Doxorubicin	Cytoreductive surgery	0.3296	0.2516
Doxorubicin	Local ablative therapy	0.6526	0.5957
Doxorubicin	Radiation therapy	0.4919	0.4117
Doxorubicin	Cytoreductive surgery, Local ablative therapy, or Radiation therapy	0.7979	0.9567
Doxorubicin	Cytoreductive surgery or Local ablative therapy	0.8881	0.8142
Trabectedin	Cytoreductive surgery	0.3409	0.0491*
Trabectedin	Local ablative therapy	0.4962	0.2829
Trabectedin	Radiation therapy	0.4289	0.3759
Trabectedin	Cytoreductive surgery, Local ablative therapy, or Radiation therapy	0.0342*	0.0375*
Trabectedin	Cytoreductive surgery or Local ablative therapy	0.0111*	0.0103*
Pazopanib	Cytoreductive surgery	0.2450	0.4861
Pazopanib	Local ablative therapy	0.6456	0.2900
Pazopanib	Radiation therapy	0.2089	0.4583
Pazopanib	Cytoreductive surgery, Local ablative therapy, or Radiation therapy	0.5708	0.9415
Pazopanib	Cytoreductive surgery or Local ablative therapy	0.3482	0.7819
Eribulin	Cytoreductive surgery	0.1395	0.0879
Eribulin	Local ablative therapy	0.3987	0.2237
Eribulin	Radiation therapy	0.9465	0.4264
Eribulin	Cytoreductive surgery, Local ablative therapy, or Radiation therapy	0.0515	0.0165*

Eribulin	Cytoreductive surgery or Local ablative therapy	0.0352*	0.0355*

Each comparison contrasts treatment episodes with versus without the indicated prior local-therapy history within the specified systemic therapy regimen. Combined categories indicate receipt of at least one listed modality. The Gehan–Breslow–Wilcoxon test gives greater weight to earlier events than the log-rank test. No adjustment for multiple comparisons was performed; p values should be interpreted in the exploratory context of the study. PFS, progression-free survival. *$p < 0.05$.

Table S2. Comparison of Log-Rank and Gehan–Breslow–Wilcoxon p Values for Overall Survival According to Prior Local-Therapy Category by Systemic Therapy Regimen

Systemic therapy regimen	Treatment category	P-value for OS	
		Log-rank test	Gehan–Breslow–Wilcoxon test
Doxorubicin	Cytoreductive surgery	0.0023**	0.0021**
Doxorubicin	Local ablative therapy	0.7644	0.2712
Doxorubicin	Radiation therapy	0.23	0.2833
Doxorubicin	Cytoreductive surgery, Local ablative therapy, or Radiation therapy	0.0038**	0.0013**
Doxorubicin	Cytoreductive surgery or Local ablative therapy	0.0005***	0.0003***
Trabectedin	Cytoreductive surgery	0.1915	0.2071
Trabectedin	Local ablative therapy	0.0832	0.5833
Trabectedin	Radiation therapy	0.4654	0.3969
Trabectedin	Cytoreductive surgery, Local ablative therapy, or Radiation therapy	0.1285	0.1059
Trabectedin	Cytoreductive surgery or Local ablative therapy	0.137	0.1007
Pazopanib	Cytoreductive surgery	0.4946	0.3146
Pazopanib	Local ablative therapy	0.9357	0.2508
Pazopanib	Radiation therapy	0.9731	0.6001
Pazopanib	Cytoreductive surgery, Local ablative therapy, or Radiation therapy	0.321	0.4646
Pazopanib	Cytoreductive surgery or Local ablative therapy	0.2885	0.1072
Eribulin	Cytoreductive surgery	0.0128*	0.0148*
Eribulin	Local ablative therapy	0.4506	0.837

Eribulin	Radiation therapy	0.7552	0.4385
Eribulin	Cytoreductive surgery, Local ablative therapy, or Radiation therapy	0.0055**	0.0037**
Eribulin	Cytoreductive surgery or Local ablative therapy	0.0044**	0.0087**

Each comparison contrasts treatment episodes with versus without the indicated prior local-therapy history within the specified systemic therapy regimen. OS was measured from initiation of the indexed systemic therapy regimen to death from any cause. Combined categories indicate receipt of at least one listed modality. The Gehan–Breslow–Wilcoxon test gives greater weight to earlier events than the log-rank test. No adjustment for multiple comparisons was performed; p values should be interpreted in the exploratory context of the study. OS, overall survival. *$p < 0.05$; **$p < 0.01$; ***$p < 0.001$; ****$p < 0.0001$.

Table S3. Univariable and Multivariable Analyses of Prognostic Factors for Progression-Free and Overall Survival in Doxorubicin-Treated Episodes

Clinicopathological factor	Progression-free survival						Overall survival					
	Univariate analysis			Multivariate analysis			Univariate analysis			Multivariate analysis		
	HR	95% CI	P-value	HR	95% CI	P-value	HR	95% CI	P-value	HR	95% CI	P-value
Age (≥65 / <65 years)	0.80	0.40–1.58	0.52				0.73	0.33-1.61	0.44			
Sex (Male / Female)	0.67	0.29–1.56	0.36				0.88	0.37-2.10	0.78			
Treatment line (2nd+ / 1st)	0.54	0.25–1.14	0.11	0.55	0.26–1.19	0.13	0.54	0.24-1.26	0.16	0.95	0.36-2.52	0.92
Performance Status (2+ / 0–1)	0.51	0.22–1.19	0.12	0.54	0.22–1.31	0.17	0.33	0.13-0.86	0.023*	0.24	0.083-0.71	0.0097**
Local therapy (Absent / Present)	1.10	0.53–2.27	0.79	1.01	0.47–2.18	0.97	0.29	0.11-0.70	0.0063**	0.22	0.079-0.60	0.0029**

Hazard ratios (HRs), 95% confidence intervals (CIs), and two-sided p values are shown for age, sex, treatment line, performance status, and local therapy. PFS and OS were measured from initiation of the indexed regimen. HRs are presented for the latter category relative to the former category listed in parentheses. Local therapy denotes receipt of at least one of the following prior local-treatment modalities: cytoreductive surgery, local ablative therapy, or radiation therapy. Each multivariable model was restricted to local therapy, included a priori as the exposure of interest, and the two additional variables with the lowest p values in the corresponding univariable analysis. Blank cells indicate variables that were not included in the corresponding multivariable model. The models were exploratory, and no adjustment for multiple comparisons was performed. HR, hazard ratio; CI, confidence interval; PFS, progression-free survival; OS, overall survival; PS, performance status. *$p < 0.05$; **$p < 0.01$; ***$p < 0.001$.

Table S4. Univariable and Multivariable Analyses of Prognostic Factors for Progression-Free and Overall Survival in Trabectedin-Treated Episodes

Clinicopathological factors	Progression-free survival						Overall survival					
	Univariate analysis			Multivariate analysis			Univariate analysis			Multivariate analysis		
	HR	95% CI	P-value	HR	95% CI	P-value	HR	95% CI	P-value	HR	95% CI	P-value
Age (≥65 / <65 years)	0.34	0.14–0.90	0.029*	0.25	0.090–0.70	0.0082**	0.74	0.24-2.25	0.59			
Sex (Male / Female)	0.92	0.33–2.55	0.87				0.52	0.15-1.88	0.32			
Treatment line (2nd+ / 1st)	0.61	0.24–1.58	0.31				0.32	0.11-0.99	0.048	0.39	0.11-1.33	0.13
Performance Status (2+ / 0–1)	0.07	0.020–0.27	0.0001***	0.05	0.011–0.20	0.0001***	0.059	0.013-0.26	0.0002***	0.09	0.018-0.48	0.0047**
Local therapy (Absent / Present)	0.26	0.072–0.99	0.048*	0.52	0.13–2.04	0.35	0.38	0.11-1.39	0.14	0.46	0.10-2.09	0.32

Hazard ratios (HRs), 95% confidence intervals (CIs), and two-sided p values are shown for age, sex, treatment line, performance status, and local therapy. PFS and OS were measured from initiation of the indexed regimen. HRs are presented for the latter category relative to the former category listed in parentheses. Local therapy denotes receipt of at least one of the following prior local-treatment modalities: cytoreductive surgery, local ablative therapy, or radiation therapy. Each multivariable model was restricted to local therapy, included a priori as the exposure of interest, and the two additional variables with the lowest p values in the corresponding univariable analysis. Blank cells indicate variables that were not included in the corresponding multivariable model. The models were exploratory, and no adjustment for multiple comparisons was performed. HR, hazard ratio; CI, confidence interval; PFS, progression-free survival; OS, overall survival; PS, performance status. *$p < 0.05$; **$p < 0.01$; ***$p < 0.001$.

Table S5. Univariable and Multivariable Analyses of Prognostic Factors for Progression-Free and Overall Survival in Pazopanib-Treated Episodes

Clinicopathological factors	Progression-free survival						Overall survival					
	Univariate analysis			Multivariate analysis			Univariate analysis			Multivariate analysis		
	HR	95% CI	P-value	HR	95% CI	P-value	HR	95% CI	P-value	HR	95% CI	P-value
Age (≥65 / <65 years)	0.60	0.29–1.22	0.16	0.59	0.29–1.21	0.15	0.45	0.18-1.12	0.09	0.31	0.11-0.83	0.020*
Sex (Male / Female)	0.74	0.37–1.47	0.39	0.79	0.39–1.59	0.51	0.98	0.44-2.19	0.96			
Treatment line (2nd+ / 1st)	0.73	0.33–1.62	0.44				0.83	0.31-2.20	0.70			
Performance Status (2+ / 0–1)	0.86	0.37–1.98	0.72				0.43	0.16-1.16	0.096	0.30	0.10-0.88	0.028*
Local therapy (Absent / Present)	0.78	0.32–1.91	0.58	0.7	0.28–1.75	0.44	0.57	0.19-1.75	0.33	0.44	0.14-1.42	0.17

Hazard ratios (HRs), 95% confidence intervals (CIs), and two-sided p values are shown for age, sex, treatment line, performance status, and local therapy. PFS and OS were measured from initiation of the indexed regimen. HRs are presented for the latter category relative to the former category listed in parentheses. Local therapy denotes receipt of at least one of the following prior local-treatment modalities: cytoreductive surgery, local ablative therapy, or radiation therapy. Each multivariable model was restricted to local therapy, included a priori as the exposure of interest, and the two additional variables with the lowest p values in the corresponding univariable analysis. Blank cells indicate variables that were not included in the corresponding multivariable model. The models were exploratory, and no adjustment for multiple comparisons was performed. HR, hazard ratio; CI, confidence interval; PFS, progression-free survival; OS, overall survival; PS, performance status. *$p < 0.05$; **$p < 0.01$; ***$p < 0.001$.

Table S6. Univariable and Multivariable Analyses of Prognostic Factors for Progression-Free and Overall Survival in Eribulin-Treated Episodes

**Clinicopathological factors**	**Progression-free survival**						**Overall survival**					
	**Univariate analysis**			**Multivariate analysis**			**Univariate analysis**			**Multivariate analysis**		
	HR	95% CI	P-value	HR	95% CI	P-value	HR	95% CI	P-value	HR	95% CI	P-value
Age (≥65 / <65 years)	0.35	0.16–0.80	0.012*	0.20	0.076–0.54	0.0013**	0.71	0.31–1.61	0.41			
Sex (Male / Female)	0.86	0.37–1.99	0.72				0.87	0.37–2.05	0.75			
Treatment line (2nd+ / 1st)	0.86	0.44–1.72	0.68				0.62	0.29–1.32	0.21	0.51	0.23–1.11	0.090
Performance Status (2+ / 0–1)	0.54	0.23–1.26	0.16	0.54	0.18–1.64	0.27	0.29	0.11–0.79	0.014*	0.36	0.12–1.15	0.084
Local therapy (Absent / Present)	0.45	0.19–1.02	0.055	0.30	0.10–0.91	0.034*	0.21	0.078–0.56	0.0020**	0.29	0.097–0.86	0.025*

Hazard ratios (HRs), 95% confidence intervals (CIs), and two-sided p values are shown for age, sex, treatment line, performance status, and local therapy. PFS and OS were measured from initiation of the indexed regimen. HRs are presented for the latter category relative to the former category listed in parentheses. Local therapy denotes receipt of at least one of the following prior local-treatment modalities: cytoreductive surgery, local ablative therapy, or radiation therapy. Each multivariable model was restricted to local therapy, included a priori as the exposure of interest, and the two additional variables with the lowest p values in the corresponding univariable analysis. Blank cells indicate variables that were not included in the corresponding multivariable model. The models were exploratory, and no adjustment for multiple comparisons was performed. HR, hazard ratio; CI, confidence interval; PFS, progression-free survival; OS, overall survival; PS, performance status. *$p < 0.05$; **$p < 0.01$; ***$p < 0.001$.

Table S7. Numbers at Risk, Events, and Follow-Up for Progression-Free Survival Analyses According to Prior Local-Therapy History by Systemic Therapy Regimen

Doxorubicin cohort (n = 44)

Local-therapy definition	Exposure group	n	PFS events	Censored	Median follow-up, months	Number at risk at month																														
						0m	0.4m	0.7m	1.2m	1.5m	1.8m	1.9m	2.1m	2.3m	2.5m	2.6m	2.7m	3.1m	3.2m	3.4m	4.4m	4.6m	4.7m	5.7m	5.8m	6.1m	6.3m	6.5m	6.9m	7.2m	8.4m	9.5m	9.6m	10.0m	22.1m	25.0m
Cytoreductive surgery	Present	24	18	6	6.5	24			24	22	21	19	18	17	16	13	12	10	9		8		7	6		5	4	3			2				1	
Cytoreductive surgery	Absent	20	17	3	NE	20	20	19	18		17	15				14		13		12		11		10	9				7	6	5	4	3	2		1
Local ablative therapy	Present	10	9	1	NE	10					10		8	7	6								4		3								2	1		
Local ablative therapy	Absent	34	26	8	22.1	34	34	33	32	29	28	26			24	23	21	19	17	16	15	14		13	11	10	9	8	7	6	5	3			2	1
Radiation therapy	Present	9	8	1	22.1	9					9							7		6					5		4				3		2		1	
Radiation therapy	Absent	35	27	8	7.2	35	35	34	33	30	29	27	25	24	23	20	18	16	15		14	13	12	11	9	8		7	6	5	4	3		2		1
Any local therapy (surgery and/or ablation and/or radiation)	Present	29	23	6	22.1	29			29	27	26	24	23	22	21	18	17	15	14	13	12		11	10	9	7	6	5			4		3	2	1	
Any local therapy (surgery and/or ablation and/or radiation)	Absent	15	12	3	NE	15	15	14	13		12	10				9		8				7		6					5	4	3	2				1
Cytoreductive surgery and/or local ablative therapy	Present	27	21	6	22.1	27			27	25	24	22	21	20	19	16	15	13	12		11		10	9	8	7	6	5			4		3	2	1	
Cytoreductive surgery and/or local ablative therapy	Absent	17	14	3	NE	17	17	16	15		14	12				11		10		9		8		7	6				5	4	3	2				1

Trabectedin cohort (n = 31)

Local-therapy definition	Exposure group	n	PFS events	Censored	Median follow-up, months	Number at risk at month																													
						0m	0.2m	0.4m	0.7m	0.9m	1.0m	1.2m	1.5m	1.6m	1.7m	1.8m	2.0m	2.2m	2.4m	2.8m	2.9m	3.0m	3.1m	4.1m	4.3m	5.1m	7.4m	8.3m	10.2m	11.0m	14.0m	19.9m	28.1m	35.0m	39.0m
Cytoreductive surgery	Present	23	17	6	28.1	23			23	22	21		20		19	18	17	16	15	14	13		12		11	10	9	8	7	6	4	3	2	1	
Cytoreductive surgery	Absent	8	7	1	14.2	8	8	7			6	5		4								3		2											1
Local ablative therapy	Present	10	9	1	NE	10							10	9			8	7							6	5				4	3	2			1
Local ablative therapy	Absent	21	15	6	11.0	21	21	20	19	18	17	15			14	13			12	11	10	9	8	7			6	5	4	3			2	1	
Radiation therapy	Present	9	6	3	28.1	9		9			8		7				6							5			4			3			2	1	
Radiation therapy	Absent	22	18	4	NE	22	22		21	20	19	18		17	16	15		14	13	12	11	10	9		8	7		6	5	4	3	2			1
Any local therapy (surgery and/or ablation and/or radiation)	Present	27	21	6	28.1	27		27	26	25	24		23	22	21	20	19	18	17	16	15		14	13	12	11	10	9	8	7	5	4	3	2	1
Any local therapy (surgery and/or ablation and/or radiation)	Absent	4	3	1	NE	4	4				3	2										1													
Cytoreductive surgery and/or local ablative therapy	Present	25	19	6	28.1	25			25	24	23		22	21	20	19	18	17	16	15	14		13		12	11	10	9	8	7	5	4	3	2	1
Cytoreductive surgery and/or local ablative therapy	Absent	6	5	1	NE	6	6	5			4	3										2		1											

Pazopanib cohort (n = 42)

Local-therapy definition	Exposure group	n	PFS events	Censored	Median follow-up, months	Number at risk at month																													
						0m	0.4m	0.7m	1.2m	1.9m	2.0m	2.2m	2.3m	2.8m	3.1m	3.4m	3.7m	4.1m	5.0m	5.1m	6.1m	6.2m	6.3m	6.5m	7.5m	9.6m	10.0m	11.0m	13.0m	14.0m	25.0m	33.0m	36.0m	40.1m	42.5m
Cytoreductive surgery	Present	26	24	2	42.4	26	26		25		23	22	19	18	15	14		13	12		11	10		9	8	7	6	5	4	3	2		1		
Cytoreductive surgery	Absent	16	14	2	NE	16		16	15	14		13		12		11	10			9	8		7	6				5		4		3		2	1
Local ablative therapy	Present	11	9	2	42.4	11	11		10			9		8					7	6						5	4	3	2				1		
Local ablative therapy	Absent	31	29	2	NE	31		31	30	28	27	26	23	22	19	18	16	15			14	12	11	10	8			7		6	4	3		2	1
Radiation therapy	Present	17	16	1	42.4	17		17		16		15	14	13		11				10	9	8					7	6		5	4	3		2	1
Radiation therapy	Absent	25	22	3	NE	25	25		24		21	20		17	15	14	13	12	11		10		9	8	6	5		4	3	2			1		
Any local therapy (surgery and/or ablation and/or radiation)	Present	36	32	4	42.4	36	36	35	34	32	31	30	26	25	21	20		18	17	16	15	14		13	12	11	10	9	8	7	5	4	3	2	1
Any local therapy (surgery and/or ablation and/or radiation)	Absent	6	6	0	NE	6			6								5				4		3	2				1							
Cytoreductive surgery and/or local ablative therapy	Present	27	24	3	42.4	27	27		26		24	23		19	16	15		14	13	12	11	10		9	8	7	6	5	4	3	2		1		
Cytoreductive surgery and/or local ablative therapy	Absent	15	14	1	NE	15		15	14	13			12	11		10	9				8		7	6				5		4		3		2	1

Eribulin cohort (n = 38)

Local-therapy definition	Exposure group	n	PFS events	Censored	Median follow-up, months	Number at risk at month																																
						0m	0.4m	0.6m	0.7m	1.0m	1.1m	1.2m	1.5m	1.6m	1.9m	2.2m	2.3m	2.8m	2.9m	3.0m	3.8m	3.9m	4.0m	4.3m	4.7m	5.1m	5.4m	5.6m	6.2m	6.3m	6.4m	7.5m	7.7m	9.5m	12.0m	14.0m	35.0m	84.8m
Cytoreductive surgery	Present	25	21	4	84.8	25	25	24	23				21	20	19	18		17	16		15	14		13	12	11	10	9	8	7	6	5	4	3			2	1
Cytoreductive surgery	Absent	13	13	0	NE	13				13	12	11	10		8		6			5			4		3										2	1		
Local ablative therapy	Present	11	10	1	84.8	11			11						10			8							7	6				5	4	3		2		1		
Local ablative therapy	Absent	27	24	3	NE	27	27	26	25	24	23	22	21	18	17	16	15		14	13	12	11	10	9	8		7	6	5				4		3		2	1
Radiation therapy	Present	11	9	2	84.8	11								11	10		9	8	7	6					5			3		2	1							
Radiation therapy	Absent	27	25	2	6.4	27	27	26	25	23	22	21	20		17	15					14	13	12	11		10	9		8			7	6	5	4	3	2	1
Any local therapy (surgery and/or ablation and/or radiation)	Present	30	26	4	84.8	30	30	29	28				26	25	24	22	21	20	19	18	17	16		15	14	12	11	10	9	8	7	6	5	4		3	2	1
Any local therapy (surgery and/or ablation and/or radiation)	Absent	8	8	0	NE	8				8	7	6	5		3								2												1			
Cytoreductive surgery and/or local ablative therapy	Present	27	23	4	84.8	27	27	26	25				23	22	21	19		18	17		16	15		14	13	12	11	10	9	8	7	6	5	4		3	2	1
Cytoreductive surgery and/or local ablative therapy	Absent	11	11	0	NE	11				11	10	9	8		6		5			4			3		2										1			

Data are presented separately for each systemic therapy regimen and local-therapy definition. 'Present' and 'Absent' indicate the presence or absence of the specified prior local-therapy history. PFS events comprised radiologic progression according to Response Evaluation Criteria in Solid Tumors, version 1.1, or death from any cause. Median follow-up was estimated separately for each exposure group using the reverse Kaplan–Meier method. NE indicates that median follow-up was not estimable because the reverse Kaplan–Meier curve did not reach 50%. Numbers at risk are measured from initiation of the indexed systemic regimen. Blank cells indicate that the number at risk was unchanged from the preceding displayed time point and do not indicate zero. Local-therapy definitions were evaluated separately and were not mutually exclusive.

Table S8. Numbers at Risk, Deaths, and Follow-Up for Overall Survival Analyses According to Prior Local-Therapy History by Systemic Therapy Regimen

Doxorubicin cohort (n = 44)

Local-therapy definition	Exposure group	n	Deaths	Censored	Median follow-up, months	Number at risk at month																																									
						0m	0.6m	0.8m	1.6m	1.8m	2.0m	2.4m	3.2m	5.3m	5.4m	6.3m	6.6m	6.8m	7.6m	8.4m	9.2m	11.2m	11.9m	12.1m	13.7m	14.6m	15.2m	16.1m	17.6m	17.9m	20.0m	20.7m	22.7m	25.0m	26.8m	26.9m	27.2m	27.3m	29.3m	29.7m	31.9m	34.8m	39.9m	50.2m	77.2m	91.1m	92.9m
Cytoreductive surgery	Present	24	15	9	39.9	24						24	23		22				21		20				19		18	17	16	14		13	12		11			10	9	8	7	6	5	4	3	2	1
Cytoreductive surgery	Absent	20	13	7	25.0	20	20	19	18	17	15			14		13	12	11		10		9	8	7		6	5				4			3		2	1										
Local ablative therapy	Present	10	9	1	NE	10																		10	9			8		7				6			5	4	3	2	1						
Local ablative therapy	Absent	34	19	15	26.9	34	34	33	32	31	29	28	27	26	25	24	23	22	21	20	19	18	17			16	15		13		11	10	9		8	7						6	5	4	3	2	1
Radiation therapy	Present	9	5	4	50.2	9										9								8		7		6					5								4		3	2	1		
Radiation therapy	Absent	35	23	12	26.9	35	35	34	33	32	30	29	28	27	26		25	24	23	22	21	20	19		18		17		15	13	12	11		10	9	8	7	6	5	4		3				2	1
Any local therapy (surgery and/or ablation and/or radiation)	Present	29	19	10	39.9	29						29	28		27	26			25		24			23	22	21	20	19	18	16		15	14	13	12		11	10	9	8	7	6	5	4	3	2	1
Any local therapy (surgery and/or ablation and/or radiation)	Absent	15	9	6	20.0	15	15	14	13	12	10			9			8	7		6		5	4				3				2					1											
Cytoreductive surgery and/or local ablative therapy	Present	27	17	10	39.9	27						27	26		25				24		23			22	21		20	19	18	16		15	14	13	12		11	10	9	8	7	6	5	4	3	2	1
Cytoreductive surgery and/or local ablative therapy	Absent	17	11	6	20.0	17	17	16	15	14	12			11		10	9	8		7		6	5			4	3				2					1											

Trabectedin cohort (n = 31)

Local-therapy definition	Exposure group	n	Deaths	Censored	Median follow-up, months	Number at risk at month																													
						0m	0.7m	0.9m	1.0m	1.3m	1.9m	2.1m	2.6m	4.2m	4.3m	4.7m	5.7m	6.5m	6.7m	8.8m	10.2m	11.2m	12.1m	13.7m	14.2m	15.5m	20.5m	21.0m	21.8m	26.8m	27.7m	28.1m	29.0m	36.9m	49.6m
Cytoreductive surgery	Present	23	13	10	28.1	23	23	22	21				20	19	18	17	16	15		14	13		12	11		10	9	8	7	6	5	4	3	2	1
Cytoreductive surgery	Absent	8	6	2	14.2	8				8	7	6					5		4			3			2							1			
Local ablative therapy	Present	10	9	1	NE	10								10	9		8	7	6			5	4				3			2	1				
Local ablative therapy	Absent	21	10	11	21.0	21	21	20	19	18	17	16	15			14	13			12	11			10	9	8		7	6			5	3	2	1
Radiation therapy	Present	9	5	4	28.1	9			9		8				7					6			5								4	3		1	
Radiation therapy	Absent	22	14	8	21.0	22	22	21		20		19	18	17		16	15	13	12		11	10		9	8	7	6	5	4	3			2		1
Any local therapy (surgery and/or ablation and/or radiation)	Present	27	16	11	28.1	27	27	26	25		24		23	22	21	20	19	18	17	16	15	14	13	12		11	10	9	8	7	6	5	3	2	1
Any local therapy (surgery and/or ablation and/or radiation)	Absent	4	3	1	14.2	4				4		3					2								1										
Cytoreductive surgery and/or local ablative therapy	Present	25	14	11	21.8	25	25	24	23				22	21	20	19	18	17	16	15	14	13	12	11		10	9	8	7	6	5	4	3	2	1
Cytoreductive surgery and/or local ablative therapy	Absent	6	5	1	NE	6				6	5	4					3								2							1			

Pazopanib cohort (n = 42)

Local-therapy definition	Exposure group	n	Deaths	Censored	Median follow-up, months	Number at risk at month																																						
						0	1.2m	1.9m	2.2m	2.8m	2.9m	3.3m	3.4m	3.5m	4.5m	4.6m	4.7m	6.5m	6.6m	7.0m	7.6m	7.7m	8.1m	8.7m	9.2m	9.3m	9.7m	10.2m	11.9m	13.4m	14.0m	14.2m	15.9m	17.1m	19.1m	21.6m	22.4m	24.4m	26.6m	34.8m	35.2m	40.1m	43.1m	46.1m	50.4m	63.7m	86.8m	96.4m
Cytoreductive surgery	Present	26	18	8	50.4	26		26	25		24			23	22		21	20	19	18	17	16			15				14		13	12		11	10		9	8	7		6		5	4	3	2		1
Cytoreductive surgery	Absent	16	11	5	34.8	16	16			15		14	13			12							11	10		9	8	7		6			5			4				3		2					1	
Local ablative therapy	Present	11	10	1	NE	11														11	10						9			8		7					6	5	4		3		2			1		
Local ablative therapy	Absent	31	19	12	17.1	31	31	30	29	28	27	26	25	24	23	22	21	20	19			18	17	16	15	14		13	12		11		10	9	8	7				6		5		4	3		2	1
Radiation therapy	Present	17	12	5	NE	17			17	16		15	14		13	12				11							10		9		8					7			6	5		4	3		2		1	
Radiation therapy	Absent	25	17	8	17.1	25	25	24			23			22			21	20	19		18	17	16	15	14	13		12		11		10	9	8	7		6	5			4			3		2		1
Any local therapy (surgery and/or ablation and/or radiation)	Present	36	25	11	50.4	36		36	35	34	33	32	31	30	29	28	27	26	25	24	23	22			21		20		19	18	17	16		15	14	13	12	11	10	9	8	7	6	5	4	3	2	1
Any local therapy (surgery and/or ablation and/or radiation)	Absent	6	4	2	13.1	6	6																5	4		3		2					1															
Cytoreductive surgery and/or local ablative therapy	Present	27	18	9	50.4	27		27	26		25			24			23	22	21	20	19	18			17		16		15	14	13	12		11	10		9	8	7		6		5	4	3	2		1
Cytoreductive surgery and/or local ablative therapy	Absent	15	11	4	34.8	15	15			14		13	12		11	10							9	8		7		6					5			4				3		2					1	

Eribulin cohort (n = 38)

Local-therapy definition	Exposure group	n	Deaths	Censored	Median follow-up, months	Number at risk at month																																
						0m	0.4m	0.6m	0.7m	1.1m	1.4m	1.5m	1.6m	1.9m	2.4m	2.8m	3.2m	4.2m	6.6m	7.1m	7.6m	7.7m	8.8m	9.1m	9.3m	9.8m	10.2m	11.4m	13.1m	15.0m	16.5m	16.9m	18.9m	19.1m	21.7m	69.8m	84.6m	84.8m
Cytoreductive surgery	Present	25	17	8	19.1	25	25	24	23		22		21			20				19	18	17		16	15	13	12	11	10	9	8	7	6	5		3	2	1
Cytoreductive surgery	Absent	13	12	1	NE	13				13		12		11	10		8	7	6				4				3		2						1			
Local ablative therapy	Present	11	11	0	NE	11					11				10	9								8	7	6		5	4			3		2	1			
Local ablative therapy	Absent	27	18	9	18.9	27	27	26	25	24		23	22	21	20		19	18	17	15	14	13	12		11		10		8	7	6		5	4		3	2	1
Radiation therapy	Present	11	8	3	69.8	11							11		10	9			8				7			6	5			4		3		2		1		
Radiation therapy	Absent	27	21	6	19.1	27	27	26	25	24	23	22		21	20		19	18	17	16	15	14		13	12		10	9	8		6		5	4	3		2	1
Any local therapy (surgery and/or ablation and/or radiation)	Present	30	22	8	69.8	30	30	29	28		27		26		25	24			23	22	21	20	19	18	17	15	14	12	11	10	9	8	7	6	4	3	2	1
Any local therapy (surgery and/or ablation and/or radiation)	Absent	8	7	1	13.1	8				8		7		6	5		4	3	2										1									
Cytoreductive surgery and/or local ablative therapy	Present	27	19	8	69.8	27	27	26	25		24		23		22	21				20	19	18		17	16	14	13	12	11	10	9	8	7	6	4	3	2	1
Cytoreductive surgery and/or local ablative therapy	Absent	11	10	1	13.1	11				11		10		9	8		7	6	5				3				2		1									

Data are presented separately for each systemic therapy regimen and local-therapy definition. 'Present' and 'Absent' indicate the presence or absence of the specified prior local-therapy history. Median follow-up was estimated separately for each exposure group using the reverse Kaplan–Meier method. NE indicates that median follow-up was not estimable because the reverse Kaplan–Meier curve did not reach 50%. Numbers at risk are measured from initiation of the indexed systemic regimen. Blank cells indicate that the number at risk was unchanged from the preceding displayed time point and do not indicate zero. Local-therapy definitions were evaluated separately and were not mutually exclusive.
